# How does psychosocial safety climate affect safety behavior in the construction industry? A cross-level analysis

**DOI:** 10.3389/fpubh.2024.1473449

**Published:** 2024-10-28

**Authors:** Wei Zhao, Shuquan Li

**Affiliations:** School of Management Science and Engineering, Tianjin University of Finance and Economics, Tianjin, China

**Keywords:** construction industry, psychosocial safety climate, safety behavior, psychological resilience, safety-related stress

## Abstract

**Introduction:**

The unsafe work of construction workers directly contributes to frequent accidents in workplaces. However, the factors influencing the safety behavior of Chinese construction workers are not yet clear.

**Methods:**

Data from 381 construction workers were analyzed to test our hypotheses.This study aimed to investigate the impact of psychosocial safety climate (PSC) on safety behavior through a cross-level model, focusing on the mediating role of psychological resilience and the moderating effect of safety-related stress.

**Results:**

The results indicated that (1) PSC was positively associated with psychological resilience and safety behavior; (2) psychological resilience mediated the relationship between PSC and safety behavior; (3) the link between PSC and safety behavior was negatively influenced by safety-related stress; and (4) all three sub-dimensions of safety-related stress moderated the effect of PSC on safety participation.

**Discussion:**

These findings elucidate the mechanisms underlying the connection between PSC, psychological resilience, safety-related stress, and safety behavior from a multi-level perspective. Additionally, strategies for enhancing the safety behavior of construction workers were discussed.

## 1 Introduction

Safety management in the workplace has always aroused interest in enterprises. Although the safety performance of the construction industry has improved year by year, it remains one of the high-risk industries prone to accidents (1). Over the past 5 years, the number of accidents and deaths in China’s construction industry has remained at more than 680 and nearly 800 each year. Numerous issues related to construction safety have complex causes, highlighting the urgent need for improved safety management performance. Sukamani et al. (2) found that the workers’ unsafe behavior and unsafe state of programmed personal protective equipment (PPE) led to accidents. Moreover, the state of PPE depends on workers’ behavior (2). The Domino Theory further revealed that unsafe behavior was the fundamental cause of accidents (3). The systemic causal model has enriched the research perspective on the cause of accidents. The accident investigation showed that safety behavior was crucial in safety management and planning (4). Subsequently, Reason (5) combined safety behavior with the defense system of the Swiss Cheese Model. Safety behavior, as the core factor, along with other potential factors, collectively formed a complete safety system. Therefore, it is necessary to explore the psychological safety climate (PSC) and individuals’ behavior and predictors to enhance safety awareness and improve their performance.

Many studies verified the antecedent variables of safety behavior, such as physical condition (6), personal traits (7), and leadership job characteristics (8). With the deepening of the research, its focus gradually shifted toward factors. The workplaces for construction are widely distributed. The mobility of construction workers and machinery, coupled with the complexity and variability of requirements, has greatly increased the difficulty and challenge of management. The construction site, as an enclosed working environment, may have many hidden safety hazards (9). Due to the limited interaction of the organizational members with external safety resources or information, they rely more on internal communication and collaboration in their daily work, highlighting the importance of effectively promoting safety behavior and reducing accident risks. As an emerging psychosocial construct, psychosocial safety climate (PSC) refers to a specific organizational climate for the psychological health of workers, describing management support and commitment to psychological health and work stress prevention (10). Psychological safety, by contrast, refers to a shared belief that the team is safe for interpersonal risk taking (11). Although both concepts are concerned with safety in the workplace, PSC focuses on broader organizational practices, while psychological safety is more closely related to interpersonal dynamics within teams (12).

The PSC can be viewed as a higher-level resource for addressing individual demands. Based on organizational personification, employees regard the organization as a peer entity capable of both material and emotional interactions (13). The organization provides sufficient resources to employees to ensure their safety performance, such as safety initiatives, etc. (14). Employees tend to identify with the organization when they realize it recognizes their contributions. With the sense of identification, employees value the care of their managers and the assistance from their colleagues. In this situation, employees engage in altruistic exchange behaviors to reciprocate the support from the organization (15). By continuously self-motivating, employees can better fulfill their safety responsibilities (16). From a behavioral perspective, this top-down management approach is more effective in stimulating employees’ proactive behavior. For example, the safety climate based on teamwork is regarded as an essential component of the Occupational Health and Safety Management System (OHSMS) (17). Therefore, it is necessary to further explore the relationship between PSC and safety behavior.

Furthermore, few research studies have explored the underlying mechanisms behind the relationship between PSC and safety behavior. According to the Context-Process-Outcomes Model (18), the psychological process mediates the influence of context variables (e.g., organization climate) on behavior outcomes (e.g., safety behavior). As a psychological process, psychological resilience reflects an individual’s ability to maintain or enhance adaptability when facing adversity or pressure (19). On one hand, when individuals fall into difficulties, psychological resilience helps to resist the threats and rebound to a balanced state through optimism or confidence to respond to unfavorable situations (19). In addition, psychological resilience also enhances the ability of risk-taking, enabling individuals to learn from accidents and improve risk awareness, which may increase safety behavior (20). On the other hand, a strong PSC ensures that the workplace is supportive, and inclusive, and prioritizes mental health. This support can make employees feel valued and understood, which strengthens their ability to cope with stress and bounce back from challenges (21). Thus, it is plausible that psychological resilience mediates the association between PSC and safety behavior.

Construction industries are always accompanied by safety-related stress due to job demands that exceed individual resources, such as heavy workloads, standardized operating procedures, as well as job roles and interpersonal trust (22–24). Previous studies found that stress may act as a moderator in the relationship between antecedent variables and job-related behavior (e.g., (25)). According to the stress-emotion theory, high-stress leads to negative emotions such as anxiety or depression. Individual emotional fluctuations can affect their decision-making, thereby weakening the effectiveness of safety management (26). Thus, the effect of PSC on safety behavior may be varied with the different levels of safety-related stress. However, few studies addressed how safety-related stress as a boundary condition affects the safety behavior of employees in construction industries.

In summary, this study aims to set up a cross-level framework to explore the impact of psychosocial safety climate (PSC) on safety behavior, with a particular focus on the mediating role of psychological resilience. Additionally, this study will analyze how varying levels of safety-related stress moderate the relationship between PSC and safety behavior, providing valuable insights for improving workplace safety management strategies both theoretically and practically.

## 2 Literature review and hypotheses development

### 2.1 Safety behavior

Safety behavior refers to a series of activities aimed at taking responsibility for maintaining safety. Safety behavior originated from Behavior-Based Safety Theory (BBS) and was gradually recognized and promoted by the public (27). Many researchers have used the BBS theory to study the safety performance of individuals or organizations. Geller et al. (28) established guidelines for BBS assistance, including expectation setting, system management, and rule-making, ensuring that BBS assistance was effective in maintaining safety behavior. However, BBS theory overlooked the bidirectional communication between organizations and individuals and the long-term effectiveness of behavioral interventions.

The construction industry is characterized by temporality and dynamics, leading to contradictions between managers and workers (29). The safety performance of projects is usually used to represent the safety outcomes (30). In early studies, safety performance was measured by accident and casualty rate (31, 32). The limitation of this research is that these indicators of safety performance can analyze the causes or rules of accidents, but cannot play a preventive role. Therefore, Neal et al. (33) further interpreted safety performance as individual behavior related to safety, integrating this behavior with organizational attributes and individual cognition.

Safety behavior has been divided into safety compliance (SC) and safety participation (SP), which was well-known by researchers. SC is the key activity of individuals at work. It serves as the fundamental baseline for implementing safety regulations, including process standardization, following instructions, etc. SP is beyond workers’ basic responsibility, which can greatly promote performance, such as communication with managers and assisting colleagues (34). Numerous studies have shown that safety behavior is crucial for preventing accidents or injuries.

### 2.2 PSC and safety behavior

Psychosocial safety climate refers to the collective views on organizational policies, practices, and procedures aimed at safeguarding workers’ psychological health and safety, primarily influenced by senior management (16). It reflects senior management’s commitment, involvement, and consultation regarding stress prevention and conveys the management’s stance on the importance of psychological health and safety in the workplace (10). Under the guidance of organizational safety commitments, employees form a consensus on their attitudes and behaviors related to safety at work. From the perspective of safety, this paper further focuses on psychosocial safety climate as the theory of support for safety. This theory represents employees’ perception of the safety view of the organization and the effect of the organization on the health and safety of employees. The organization encourages employees with high safety performance while employees give positive feedback to the organization. The formation of collective perception climate has stability and diffusion effect (35).

The PSC can be regarded as the fit of an individual with the safety value of human resource management. Employees enhance their confidence to deal with problems through resource cognition (36). Based on the principle of reciprocity, employees will voluntarily engage in safety behavior to address risks. Managers show their concern for employees by strengthening safety supervision. Employees believe in the organization’s positive guidance of safety behavior, which increases the likelihood of complying with safety regulations or participating in safety activities (37). Lisa et al. (38) believed that employees were inclined to communicate with leadership frequently and consciously abide by safety rules after receiving material or spiritual incentives, thus preventing accidents. If there was insufficient communication or conflicting cognition, employees’ desire to participate in safety would decrease significantly, thereby affecting their initiative to deal with problems (39). Thus, the current study examined the effects of PSC on two sub-dimensions of safety behavior:

*H1a*: PSC has a positive influence on SC.

*H1b*: PSC has a positive influence on SP.

### 2.3 The mediating role of psychological resilience

From an evolutionary perspective, psychological resilience is a protective psychological mechanism formed by humans when adapting to adversity. It is a positive psychological trait with plasticity, demonstrating an individual’s ability to overcome difficulties (40). Psychological resilience develops through processes that are not shaped by natural selection but emerge as a “by-product” associated with individual characteristics. As a protective resource, PSC is strongly correlated with characteristics such as emotional commitment and a positive attitude. First of all, construction workers with resilience are more confident in handling challenging tasks successfully because they perceive that the organization provides sufficient resources for them. Amoadu et al. (41) believed that PSC was a major factor in maintaining employees’ work engagement and cultivating their collective consciousness and responsibility. Employees were keen to participate in activities actively and tended to achieve their goals accordingly. Second, PSC convinces employees that the organization will attempt remedial measures to minimize losses after accidents. Even if employees encounter difficulties, they can quickly find solutions with the help of the organization. Employees learn from the experience of accidents, enhance their safety skills, and achieve the spiral improvement of resilience. Managers can construct a psychological safety climate for employees from interaction and cultural perspectives (42). Therefore, PSC may have a positive influence on psychological resilience.

In addition, Bandura (43) mentioned in social cognition theory that individual behavior was not only driven by cognitive or emotional factors but also shaped by organizational contexts. Psychological functioning involves the interaction of traits, behavior, and environment, which helps individuals deal with emergencies calmly and control their violations (44). In the construction industry, workers more sensitive to safety are likely to abide strictly by regulations. Even in poorly regulated areas, workers try to regulate unsafe behavior to avoid accidents. McCabe et al. (20) found that individual resilience can alleviate the psychological stress of Canadian construction workers and correlate with safety performance. Workers with stronger resilience followed the guidance of the managers modestly after accidents. They learned from the experience and corrected their behavior to prevent similar accidents from happening again. Xu et al. (45) conducted comparative experiments on safety climate intervention. The results showed that organizational resources promoted safety motivation. Employees relieved their burnout, which promoted safe behavior accordingly. Thus, we hypothesized the following:

*H2a*: Psychological resilience mediates the relationship between PSC and SC.

*H2b*: Psychological resilience mediates the relationship between PSC and SP.

### 2.4 The moderating role of safety-related stress

Enterprises tend to gain benefits with fewer resources. Therefore, employees face higher job demands. If employees are exposed to high-intensity physical or mental labor for a long time, it can trigger stress responses. Safety-related stress is considered to arise from constraints, indicating that an individual’s resources are insufficient for job demands (46). In this case, the departure from the work goal may compromise safety performance. Sampson et al. (47) described safety-related stress based on occupational stress, including safety-role ambiguity, safety-role conflict, and interpersonal safety conflict. Safety-role ambiguity refers to employees’ unclear understanding of their responsibilities because of inadequate information (48). Employees were confused about behavioral decisions, leading to a lack of confidence. Safety-role conflict was considered to be inconsistent between employees’ expectations of job performance and others’ evaluations (49). When receiving different or conflicting orders, employees were trapped in a dilemma and engaged in dangerous behaviors. Interpersonal safety conflict arose when there were disagreements with other members. There was a correlation between interpersonal safety conflict and prosocial safety behavior, which limited employees’ participation in safety management (50). According to the job demand-resource theory, the employees’ physical or psychological stress is induced by the high demands, which weakens their problem-solving ability and negatively affects individual behavior (51).

When the PSC level is low, long-term job demands often lead to a lack of channels to effectively report work overload and fatigue within the organization. Nevertheless, PSC is largely driven by the values and beliefs about management. Unfortunately, managers often underestimate the role of PSC in maintaining employees’ mental health and productivity, which elicits employees’ stress (52). When safety-related stress is low, employees are more likely to view the organization’s PSC as effective and supportive, enhancing their trust in management and motivating them to engage in safe behaviors (53). They can better utilize cognitive and emotional resources, focusing on understanding and implementing safety protocols, participating in safety training, and contributing to a safe work environment. Conversely, when safety-related stress is high, even the organization’s efforts to create a safe climate may be overshadowed by this persistent stress, causing employees to feel unsupported and skeptical about the effectiveness of safety measures, thereby reducing their compliance and participation in safety behaviors (54). High stress depletes employees’ cognitive and emotional resources, making it difficult for them to engage with PSC initiatives, leading to burnout, fatigue, and decreased attention to safety details, which weakens the positive impact of PSC on safety behavior (55). Thus, the following hypotheses are proposed:

*H3a*: Safety-related stress moderates the relationships between PSC and SC.

*H3b*: Safety-related stress moderates the relationships between PSC and SP.

*H4*: Three sub-dimensions of safety-related stress, namely: safety role ambiguity (H4a), safety role conflict (H4b) and interpersonal safety conflict (H4c) moderate the relationship between PSC and SC.

*H5*: Three sub-dimensions of safety-related stress, namely: safety role ambiguity (H5a), safety role conflict (H5b) and interpersonal safety conflict (H5c) moderate the relationship between PSC and SP.

**Figure 1:**
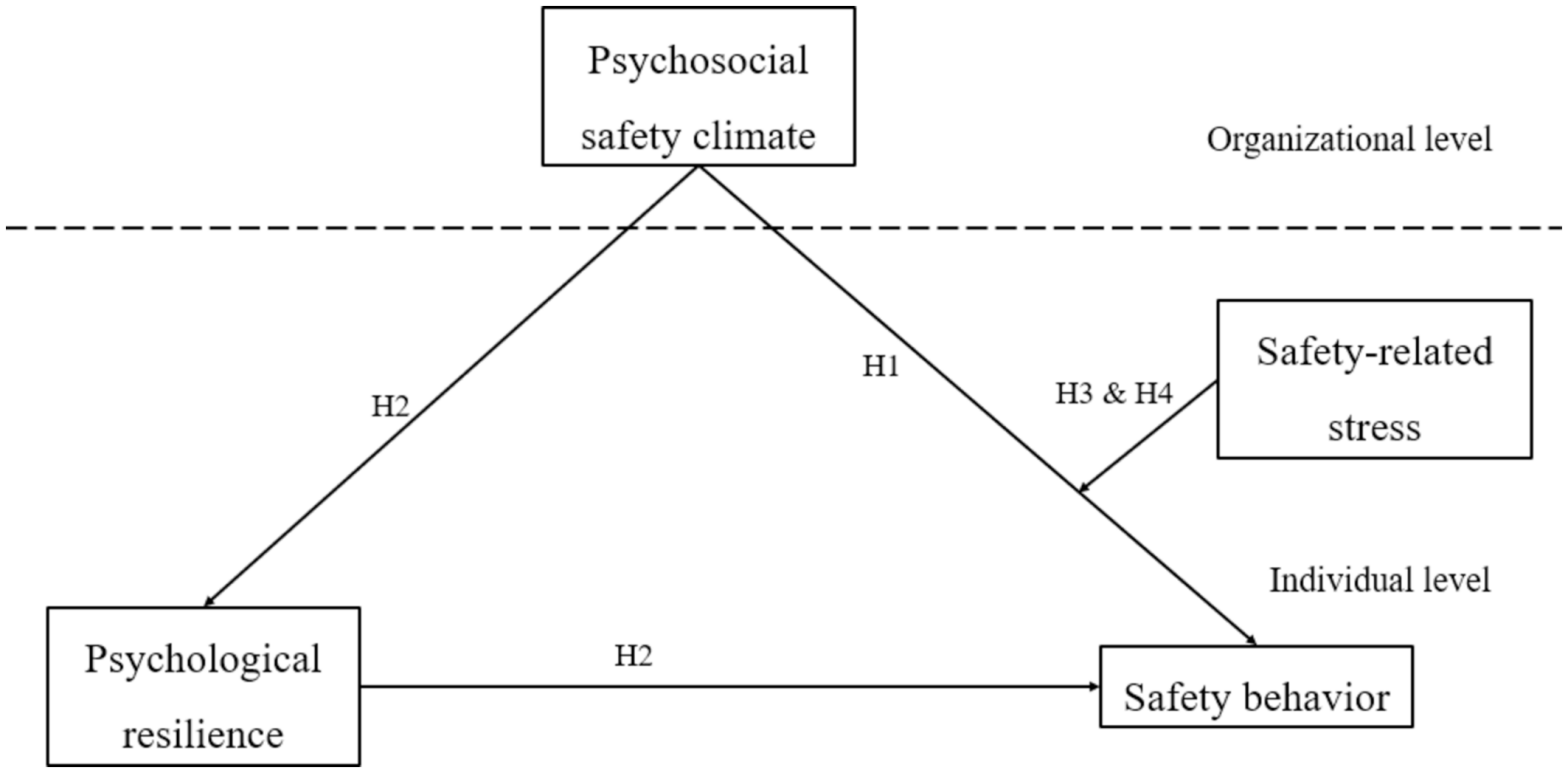
Conceptual model.

Based on the literature reviewed, to elucidate the underlying mechanism between PSC and safety behavior, the present study tested the impact of PSC on safety behavior the mediating role of psychological resilience, and the moderating role of safety-related stress in this relationship. Based on the interrelationships among the above variables, the conceptual model is shown in Figure 1.

## 3 Materials and methods

### 3.1 Participants

The research samples were collected from workers in the construction industry. The formal investigation was conducted on 11 construction projects in Tianjin City and Hebei Province in China. After the data cleaning (e.g., Questionnaires with more than 25% missing data were considered invalid and removed from the dataset), a total of 381 construction workers participated in the survey. Table 1 presents the demographic information of the participants.

**Table 1:** Demographic data for participants.

Category	Item	*N*	Percentage (%)
Gender	Male	361	94.7
	Female	20	5.3
Age	18–30	97	25.5
	31–40	130	34.1
	41–50	126	33.2
	≥51	28	7.2
Education level	Primary school	43	11.2
	Junior high school	158	41.4
	High school	113	29.7
	College degree or above	67	17.7
Work tenure	≤5	97	25.5
	6–10	121	31.7
	11–15	69	18.2
	≥16	94	24.6
Type of work	Reinforced worker	101	26.5
	Carpenter	87	22.8
	Mason	32	8.4
	Concrete worker	24	6.2
	Shelf worker	16	4.2
	Electrician	16	4.2
	Other	105	27.7

### 3.2 Procedures

All participants were informed in advance about the purpose and procedures of the survey to ensure they understood the significance of the research activities. One or two managers were entrusted to explain the items in the questionnaire so that participants understood the items more accurately. In addition, managers acted as supervisors during the data collection, ensuring that the entire procedure complied with the investigation procedures. The participants were asked to respond anonymously to alleviate any concerns. During the survey, we emphasized the ethicality of the activity to the participants, stating, for instance, that the data would only be used for academic research purposes.

To reduce common method variance, a two-stage collection was adopted for data investigation. In the first stage, we gathered demographic information and sent questionnaires to workers about the psychosocial safety climate. Three weeks later, questionnaires were distributed to the participants who responded effectively in the first stage to rate the psychological resilience, safety-related stress, and safety behavior.

### 3.3 Measures

The questionnaire consisted of individual information and items for each variable adopted from previous studies. Some of the items were modified to make them suitable for the study. For all measurements, responses were collected using a five-point Likert scale ranging from 1 (strongly disagree) to 5 (strongly agree).

#### 3.3.1 Psychosocial safety climate

The psychosocial safety climate was evaluated by the 12-item scale (PSC-12) developed by Hall et al. (56) and Li et al. (57) revised it to the Chinese version. Sample items included “Senior management acts decisively when a concern over an employee’s psychological status is raised” and “Information about workplace psychological well-being is always brought to my attention by senior management.” The Cronbach’s *α* of this scale is 0.91.

#### 3.3.2 Psychological resilience

Workers rated psychological resilience using a 6-item scale from Smith et al. (58). The Chinese version of this scale has demonstrated acceptable internal consistency with Cronbach’s alpha values 0.72 in mainland samples (59). An example item was “I can quickly recover from the panic of the accident.” The Cronbach’s *α* of this scale is 0.84.

#### 3.3.3 Safety behavior

Safety behavior is measured using the questionnaire developed by Griffin and Neal (33), comprising 4 items for safety compliance and 5 items for safety participation. This scale has demonstrated acceptable internal consistency in Chinese population (60). Sample items included “I will follow the operating procedures and use the necessary safety equipment” and “I will make extra efforts to ensure a safety workplace.” The Cronbach’s *α* of this scale is 0.87.

#### 3.3.4 Safety-related stress

The safety-related stress was evaluated using a 13-item scale developed by Sampson et al. (47), consisting of 3 dimensions (i.e., safety role ambiguity, safety role conflict, and interpersonal safety conflict). This scale was adapted into Chinese by Wang et al. (60). Sample items included “I work without clear, planned safety goals,” “I worked in more than two teams with different safety practices” and “I have argued with others about safety at work.” The Cronbach’s *α* of this scale is 0.86.

#### 3.3.5 Control variables

Age, education level, and work tenure were controlled at the individual level. Because previous studies have suggested that the three characteristics may influence safety behavior (61, 62). Besides, we controlled for organizational size and tenure when evaluating the organizational level.

### 3.4 Statistical analysis

Since safety behavior is affected by factors such as individuals and organizations, a cross-level model is a more suitable analytical tool. We decided to use Mplus 8 for path analysis about previous studies to test the validity of the above hypotheses (63). Specifically, when using a cross-level model for estimation, it usually starts with the null model, which constructs a model without any independent variables to examine whether the variance of the dependent variable in the first layer is significant between the second layer. If significant, it is suitable for further analysis. The cross-level model is suitable for processing nested structured data to reveal causal relationships between variables in multi-layer nested structures.

## 4 Results

### 4.1 Data aggregation

To evaluate the viability of aggregating the individual-level data of each construction worker on PSC to the organizational-level, we examined both between-groups variation and within-groups agreement. This study used inter-group correlation coefficients ICC (1), ICC (2), and intra-group consistency coefficient *R*_wg_ as indicators for evaluating data aggregation. ICC (1) is generally considered to indicate whether there is a significant inter-group difference between different groups, and its value should be greater than 0.05. ICC (2) represents reliability of mean score at the organizational level, and its value should be greater than 0.7 (64). *R*_wg_ represents the degree of consistency in member responses, and its value should be greater than 0.7 (65). The results showed that the values of *R*_wg_, ICC (1), and ICC (2) were 0.92, 0.21, and 0.75, respectively. Therefore, the data of PSC measured at the individual level can be aggregated to the organizational level.

### 4.2 Confirmatory factor analysis

In this study, the Harman single factor method was adopted to test the common method variance of the variables. The principal component analysis without rotation was conducted for all the items (66). The results showed that the total variance explained by the first factor is 26.62%, which is lower than 40%. In addition, this study employed confirmatory factor analysis to evaluate the discriminant validity of core variables. According to Table 2, the results of the four-factor model fit the data well (*χ*^2^/df = 1.463, RMSEA = 0.036, TLI = 0.935, CFI = 0.932, SRMR = 0.042). Besides, this result is significantly superior to other multi-factor or single-factor competition models, indicating that the variables designed in this study have acceptable discriminant validity.

**Table 2:** Results of confirmatory factor analysis.

Model	*χ*^2^	*χ*^2^/df	RMSEA	TLI	CFI	SRMR
Four-factor model: A, B, C, D	1355.014	1.463	0.036	0.935	0.932	0.042
Three-factor model: A + B, C, D	2499.258	2.675	0.054	0.872	0.866	0.071
Three-factor model: A, B + C, D	2723.106	2.903	0.049	0.896	0.869	0.070
Two-factor model: A + B + C, D	5223.150	5.529	0.076	0.775	0.814	0.095
One-factor model: A + B + C + D	7835.724	8.282	0.087	0.573	0.672	0.122

*N = 381. A, psychosocial safety climate; B, psychological resilience; C, safety-related stress; D, safety behavior.*

### 4.3 Descriptive statistics

Table 3 shows the means, standard deviations and correlations of all the focal variables. Psychological resilience is significantly positively correlated with safety behavior (*r* = 0.454, *p* < 0.01), while has a negative association with safety-related stress (*r* = −0.318, *p* < 0.01). Safety behavior is negatively related to safety-related stress (*r* = −0.372, *p* < 0.01).

**Table 3:** Means, standard deviations, and correlations of variables.

Variables	Mean	SD	1	2	3	4	5
Individual level							
1. Age	41.557	11.642					
2. Education	1.942	2.568	−0.152^**^				
3. Work tenure	1.956	8.741	0.678^**^	−0.185^**^			
4. Psychological resilience	3.452	1.022	0.063	0.062	0.070		
5. Safety behavior	3.598	1.144	0.026	0.057	0.103	0.454^**^	
6. Safety-related stress	3.157	0.983	−0.054	−0.039	−0.094^**^	−0.318^**^	−0.372^**^
Organizational level							
1. Organizational size	2.446	0.503					
2. Organizational tenure	2.178	0.588	0.132^*^				
3. Psychosocial safety climate	3.568	0.869	0.106^*^	0.080			

*N* = 381; ^*^p < 0.05, ^**^p < 0.01.

### 4.4 Hypothesis testing

Before cross-level analysis, psychological resilience and safety behavior were set as output variables, and then the null model was tested. The results showed that ICC (1) were 0.21 and 0.13, respectively (*p* < 0.01), both greater than 0.06. Therefore, it is necessary to use a hierarchical linear model for analysis.

Models 2 and 7 illustrated the significant effects of PSC on SC (*β* = 0.24, *p* < 0.01) and SP (*β* = 0.35, *p* < 0.01), which supported H1a and H1b. According to model 1 in Table 4, PSC influenced psychological resilience positively (*β* = 0.28, *p* < 0.05). The results of models 3 and 8 showed that psychological resilience was positively correlated with SC (*β* = 0.26, *p* < 0.01) and SP (*β* = 0.29, *p* < 0.01), respectively. The next step was conducted by introducing psychological resilience to test the mediation effect. We found that the relationship between PSC and SC (*β* = 0.18, *p* < 0.01) or SP (*β* = 0.29, *p* < 0.01) remained significant. The decrease in the regression coefficient indicated that psychological resilience partially mediated the relationship between PSC and SC and SP. Additionally, we used Monte Carlo-based simulation (10,000 replications), i.e., parametric bootstrapping method, to further evaluate the indirect effects. The results showed that the bias-corrected 95% confidence intervals for the indirect effects of SC and SP were (0.05, 0.16, 0.09, 0.23), both excluded 0, thereby supporting H2a and H2b.

**Table 4:** Regression analysis results.

Variable	PR	Safety compliance (SC)					Safety participation (SP)				
	M 1	M 2	M 3	M 4	M 5	M 6	M 7	M 8	M 9	M 10	M 11
Intercept	4.03^**^	7.18^**^	7.42^**^	7.93^**^	8.18^**^	8.55^**^	10.33^**^	11.65^**^	13.81^**^	15.63^**^	15.98^**^
Organizational level											
Organizational size	0.01	0.01^*^	0.01^*^	0.01^*^	0.01^*^	0.02^*^	0.01	0.01	0.02	0.02	0.02
Organizational tenure	0.02	0.01	0.02	0.02	0.03	0.03	0.02	0.02	0.02	0.03	0.02
PSC	0.28^*^	0.24^**^		0.18^**^	0.19^**^	0.18^**^	0.35^**^		0.29^**^	0.27^**^	0.24^**^
Individual level											
Age	−0.01	0.01	0.01	0.01	0.00	0.00	0.01	0.01	0.01	0.01	0.00
Education	−0.02	0.02	0.02	0.01	0.01	0.01	0.02	0.01	0.01	0.00	0.00
Work tenure	−0.03	0.03^*^	0.02^*^	0.02^*^	0.02^*^	0.02^*^	0.04^*^	0.03^*^	0.03^*^	0.03^*^	0.02^*^
PR			0.26^**^	0.17^*^				0.29^**^	0.24^**^		
SS					−0.16^*^	−0.11^*^				−0.19^*^	−0.12^*^
Cross-level interaction											
PSC × SS						−0.12^**^					−0.14^**^
PSC × SRA						−0.13^**^					−0.18^**^
PSC × SRC						−0.05					−0.16^**^
PSC × ISC						−0.07					−0.13^*^
Deviance	594.31	677.47	667.43	659.46	652.83	646.38	557.11	554.45	551.20	545.27	540.22

*N* = 381; ^*^p < 0.05, ^**^p < 0.01; M, model; PSC, psychosocial safety climate; PR, psychological resilience; SS, safety-related stress; SRA, safety role ambiguity; SRC, safety role conflict; ISC, interpersonal safety conflict.

Meanwhile, models 6 and 11 indicated that the moderating effect of safety-related stress on the relationship between PSC and SC (*β* = −0.12, *p* < 0.01) and SP (*β* = −0.14, *p* < 0.01) was significant, so H3 were supported. The results also suggested that safety role ambiguity moderated the relationship between PSC and SC negatively (*β* = −0.13, *p* < 0.05), but the other two sub-dimensions had no significant moderating effect, hence supporting H4a. Moreover, all the sub-dimensions played a moderating role in the PSC-to-SP relationship (safety role ambiguity: *β* = −0.18, *p* < 0.01; safety role conflict: *β* = −0.16, *p* < 0.01; interpersonal safety conflict: *β* = −0.13, *p* < 0.05), respectively. Thus, H5 was supported.

**Figure 2:**
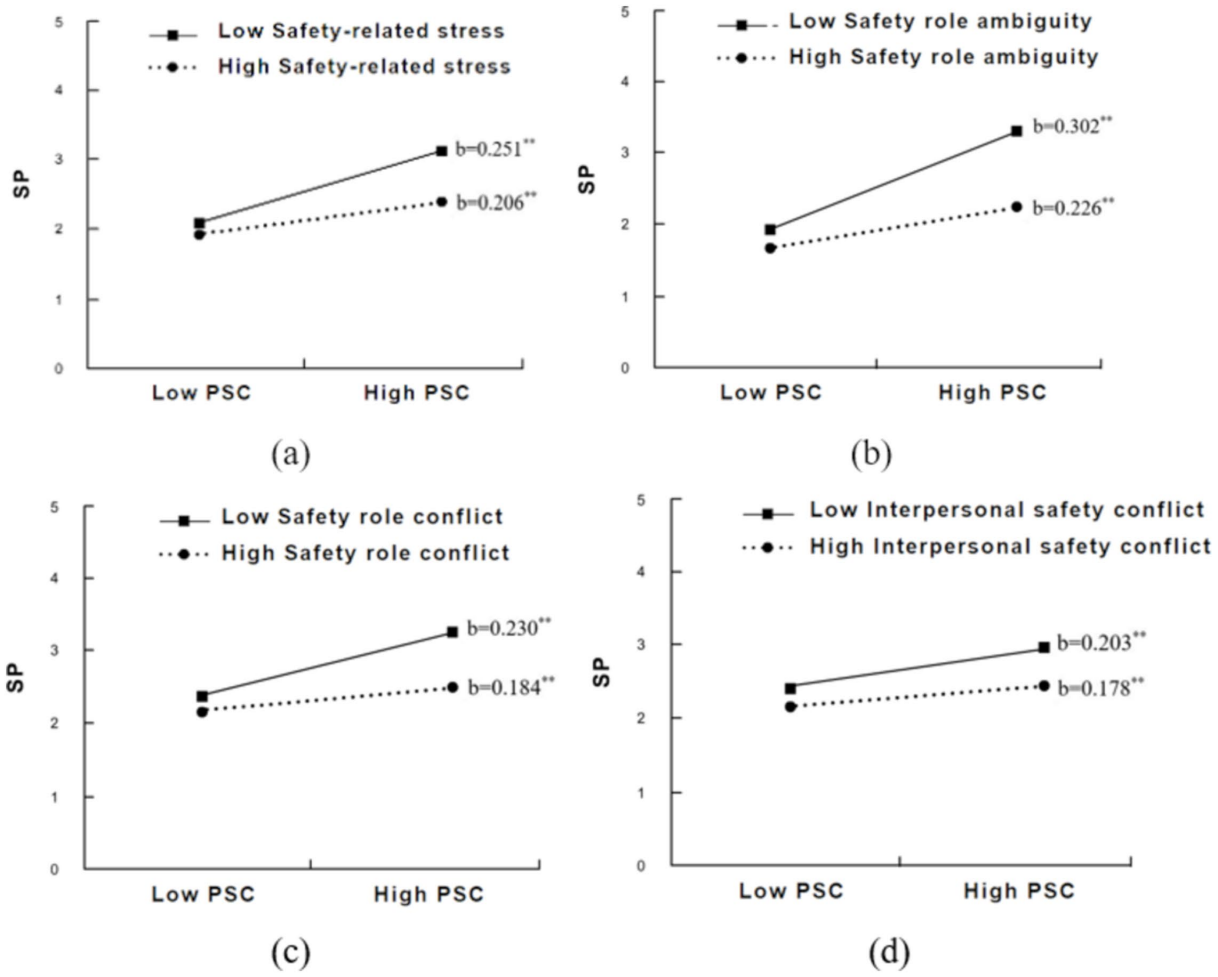
Moderating effect of safety-related stress and its sub-dimensions on the relationship between PSC and SP. PSC = psychosocial safety climate, SP = safety participation. **(a)** safety-related stress; **(b)** safety role ambiguity; **(c)** safety role conflict; **(d)** interpersonal safety conflict Note. *p < 0.05, **p < 0.01.

The slope charts of the moderating effect of safety-related stress and its three sub-dimensions on the relationship between PSC and SP are shown in Figure 2. The results further confirmed the conclusions above. Compared to the workers with lower safety-related stress, the simple slope relating PSC to SP at higher safety-related stress is much less. In other words, PSC had a stronger impact on workers with lower safety-related stress.

## 5 Discussion

This study constructed a cross-level model to explore the impact of the interaction between PSC and safety-related stress on workers’ safety behavior in the construction industry, mediated by psychological resilience. Specifically, PSC can directly promote safety behavior, and can also indirectly influence safety behavior through psychological resilience. In addition, safety-related stress moderates the relationship between PSC and safety behavior. Specifically, for workers with lower stress, PSC can better improve their behavior. This is because lower stress can better stimulate workers’ safety awareness and motivation, so they tend to adopt more prudent strategies to complete their work (67).

### 5.1 Theoretical implications

The research findings have yielded several theoretical contributions. First, we found that PSC was significantly associated with SC and SP. In the current study, we have enriched the concept of safety support as an organizational-level variable and expanded its research scope. Based on the research of Newaz et al. (68), this paper further underscores the key role of PSC in individual spontaneous behavior. These conclusions support the antecedents of safety behavior. Similar to the findings of Rhoades et al. (69), the provision of supportive resources by an organization significantly enhances construction workers’ enthusiasm and engagement. Employees are not only dedicated to their work but also check safety vulnerabilities in the workflow from a comprehensive perspective. They are motivated to become more involved in safety activities by providing improvement suggestions to their colleagues or superiors (70). Consistent with Smith’s et al. (71) research, managers should seek leadership strategies to enhance workers’ perception of safety climate. The psychosocial safety climate can let employee recognitive that safety is prioritized over production (72), which is crucial for the development of workers’ safety habits. Furthermore, the conclusions extended He et al.’s (73) findings. Workers are inclined to prioritize tasks within their responsibilities without sufficient supportive resources, such as complying with safety rules and avoiding transgressions. Accordingly, the resources investment can be viewed as a potential strategy to enhance workers’ engagement in safety activities.

Second, from the perspective of individual characteristics, this study revealed the mediating role of psychological resilience in the relationship between PSC and safety behavior. Namely, the organization has established a management position of safety values and priorities at the construction site, which greatly improves the workers’ confidence in facing dangerous situations. PSC ensures that employees believe the organization will take remedial measures to minimize losses after accidents. This belief helps employees quickly find solutions during difficulties, learn from accidents, enhance their safety skills, and progressively improve their resilience (74). In addition, individuals with higher resilience will react more calmly and solve problems reasonably when encountering emergencies (75). Moreover, Individuals with higher resilience are more likely to view accidents as a challenge. They are good at learning from failures and gradually improving their solutions (76). In high-pressure situations, employees with high psychological resilience are less likely to experience overwhelming anxiety, allowing them to remain calm and make rational decisions, which is crucial for maintaining safety protocols and responding appropriately to hazards (77). Furthermore, employees also perceive senior management support and specific regulations as the main drivers of PSC, which has a positive impact on their mental health (78). The organization’s attitude toward handling employees’ errors can also influence their psychological outcomes. For example, the psychological safety of employees can be enhanced by error management (79). In summary, underscoring the mediating role of psychological resilience, this framework explores the underlying psychological mechanisms by which PSC cultivates workers’ safety behavior. The findings enhance our understanding of the psychosocial factors that influence workers’ safety behavior and provide theoretical insights to clarify this relationship.

Third, previous research on the moderating effect of safety-related stress on safety behavior seemed insufficient. Therefore, the current study considers safety-related stress as a boundary condition, highlighting the importance of stress differences in altering the effects of PSC on safety behavior. According to the conservation of resource theory, the mismatch between the supply and consumption of resources leads to unsafe behavior. When job demands exceed available resources, individuals may experience negative emotions such as panic or anxiety, further affecting behavioral decision-making (80). Moreover, despite individual perceptions, stress in the workplace appears to be inevitable (81). Therefore, it is essential to explain behavioral cognition in the stress climates. In this study, all three sub-dimensions of safety-related stress had significant moderating effects on the relationship of PSC-SP. Contrary to the hypotheses, the moderating effect of safety role conflict and interpersonal safety conflict in the PSC-SC relationship was insignificant. The possible explanation may be that due to the lack of safety awareness and skills, construction workers are used to completing tasks following the safety regulations, so they are not willing to participate in safety activities beyond the basic requirements of work. Mohsen et al. (82) also made similar conclusions in the survey of industrial organizational employees, highlighting the significant role of work stress in moderating employees’ emotion and behavior. Employees are more willing to participate in organizational activities with low stress, that is, showing organizational citizenship behavior. Moreover, safety role ambiguity played a moderating role in the PCS-to-safety behavior’s sub-dimensions relationship, which was also a valuable response to previous studies (83, 84). The mechanism of role ambiguity also applied in the safety domains. The above implications extend the theoretical understanding for safety behavior guidance.

Overall, we construct a multilevel model to expound how PSC permeates the organizational hierarchy to drive individual behavior, and extend cross-level research through psychological resilience and safety-related stress. The traditional approach ignores the fact that PSC and individual-related variables at different levels. From an organizational level perspective, we can add a more reliable understanding of PSC. In this study, we find a top-down relationship between PSC and safety behavior. Therefore, the cross-level model is considered to be more suitable for the approach in this paper.

### 5.2 Practical implications

The PSC not only directly affects the safety behavior of construction workers, but also has an indirect effect on safety behavior through psychological resilience. Thus, interventions for safety behavior can be made by improving workplace climate and psychological resilience. Managers must hold safety training regularly for workers due to the lack of safety knowledge and the oversight of safety supervision by enterprises (85). Workers have much immediate decision-making in their working process with high uncertainty (86). Consequently, when faced with job demands that are beyond their responsibilities, workers find it challenging to follow the safety procedures, leading to numerous hazards. Managers need to improve risk prevention by ensuring that unequivocal danger signals are transmitted to workers in real-time so that they can make behavioral predictions based on specific situations. Previous research has confirmed that for small and medium-sized enterprises (SMEs) with insufficient safety budgets, employees ensured the tasks could be completed successfully through informal workflows (87). Furthermore, a high accident rate can adversely affect the psychological health of construction workers, leading to emotional distress or interpersonal conflicts. Workers with stronger resilience are more adaptable to poor working settings, showing a strong desire to communicate with managers. The organization can timely understand the demands of workers, thereby making targeted improvements to working contexts (88). Workers motivate themselves to keep learning and improve skills when perceiving that the organization values their safety intentions. Some studies showed that many occupational safety hazards can be better controlled with strict management (89). Owing to advanced equipment and protective devices, workers’ safety performance in the workplace has dramatically improved. Managers should provide praise to workers with outstanding safety performance. It will develop workers’ self-identity and contribute to creating a safe climate within the organization.

Considering the negative moderating effect of safety-related stress, managers should realize that reducing stress is a way to improve construction workers’ safety behavior. Safety role ambiguity is the most significant predictor among the sub-dimensions of safety-related stress (60). Workers sometimes encounter difficulties in comprehending their responsibilities and authorities, which may be due to disorganized scheduling or inadequate training (90). During the project, it’s essential to ensure a clear delineation of responsibilities within the organization. Workers internalize their safety role positioning, incorporating safety expectations into their daily tasks (60). Besides, managers try to create an open communication platform for workers. When there is a disagreement with workers, it is necessary to ensure that their opinions are given full attention and feedback. Finally, managers should be aware of cultivating interpersonal relationships among workers. Workers build a solid trust foundation through formal or informal interactions, showing stronger resilience in emergencies (91).

### 5.3 Limitations and future research directions

Despite these contributions, this research has several limitations. First, the study adopted a cross-sectional design due to time or capital costs. Safety-related stress focuses on an individual’s response to stress, which means it is necessary to evaluate the negative effect over a while. Therefore, we suggest employing a longitudinal design to evaluate causal relationships among the variables in the future. In addition, more individual or organizational characteristics served as mediators or moderators should be adopted to optimize the model, such as leadership style, emotional intelligence, etc., further uncovering how PSC influences safety behavior. In previous studies, the measurement of PSC contained single or multi-dimensional construct. Hence, a more comprehensive scale should be further developed based on the scale according to the standardized development. Finally, the participants were from a few construction projects, which may affect the model’s applicability. Subsequent research should involve cross-industry samples to validate the model and consolidate the findings of this study.

## 6 Conclusion

In this study, we construct a cross-level model to examine how psychosocial safety climate affects safety behavior. The results indicate that PSC can promote workers’ safety behavior through psychological resilience. Additionally, this paper attempts to explain the relationship between PSC and safety behavior from the stress perspective, which identifies a significant moderating effect of safety role ambiguity. Therefore, managers can formulate interventions based on the findings of this study, particularly for improving the workers’ safety behavior in construction industries.

## Data Availability

The raw data supporting the conclusions of this article will be made available by the authors, without undue reservation.
